# Molecular Genetics External Quality Assessment Pilot Scheme for Irinotecan-Related *UGT1A1* Genotyping in China

**DOI:** 10.1371/journal.pone.0148081

**Published:** 2016-01-28

**Authors:** Lang Yi, Guigao Lin, Kuo Zhang, Lunan Wang, Rui Zhang, Jiehong Xie, Jinming Li

**Affiliations:** 1 National Center for Clinical Laboratories, Beijing Hospital, Beijing 100730, P R China; 2 Graduate School, Peking Union Medical College, Chinese Academy of Medical Sciences, Beijing 100730, P R China; Centro di Riferimento Oncologico, IRCCS National Cancer Institute, ITALY

## Abstract

Irinotecan is widely used in the treatment of solid tumors, especially in colorectal cancer and lung cancer. Molecular testing for *UGT1A1* genotyping is increasingly required in China for optimum irinotecan administration. In order to determine the performance of laboratories with regard to the whole testing process for *UGT1A1* to ensure the consistency and accuracy of the test results, the National Center for Clinical Laboratories conducted an external quality assessment program for UGT1A1*28 genotyping in 2015. The panel, which comprised of four known mutational samples and six wild-type samples, was distributed to 45 laboratories that test for the presence of UGT1A1*28 polymorphisms. Participating laboratories were allowed to perform polymorphism analysis by using their routine methods. The accuracy of the genotyping and reporting of results was analyzed. Other information from the individual laboratories, including the number of samples tested each month, accreditation/certification status, and test methodology, was reviewed. Forty-four of the 45 participants reported the correct results for all samples. There was only one genotyping error, with a corresponding analytical sensitivity of 99.44% (179/180 challenges; 95% confidence interval: 96.94−99.99%) and an analytical specificity of 100% (270/270 challenges; 95% confidence interval: 98.64−100%). Both commercial kits and laboratory development tests were commonly used by the laboratories, and pyrosequencing was the main methodology used (n = 26, 57.8%). The style of the written reports showed large variation, and many reports showed a shortage of information. In summary, the first *UGT1A1* genotyping external quality assessment result demonstrated that *UGT1A1* genotype analysis of good quality was performed in the majority of pharmacogenetic testing centers that were investigated. However, greater education on the reporting of *UGT1A1* genetic testing results is needed.

## Introduction

Irinotecan (CPT-11, Camptosar), an anticancer drug that inhibits topoisomerase I, is frequently used as a standard first-line treatment for advanced colorectal cancer. Furthermore, the drug is used to treat a range of other cancers, including lung cancer, gastric cancer, and gynecologic neoplasms [1–4]. However, its application is limited because of interindividual differences in severe toxicity reactions such as diarrhea and neutropenia [5,6]. Irinotecan is a prodrug, and the enzyme uridine diphosphate glucuronosyltransferase (UGT1A1) is responsible for the inactivation of irinotecan’s active metabolism. It has been well documented that genetic polymorphisms of *UGT1A1*, such as UGT1A1*28, cause reduced enzymatic activity; therefore, they are considered to be predictive markers of irinotecan-related toxicity [7–9]. However, some such conclusions are still controversial because of the distinct differences in the frequency of the *UGT1A1* genotype between western and eastern countries [10,11]. Since UGT1A1*28 homozygous individuals have only 35% of the activity in wild-type *UGT1A1* individuals and metabolize irinotecan more slowly [10,12], cancer patients with the UGT1A1*28/*28 genotype are at an increased risk of high-grade neutropenia and/or diarrhea while being treated with irinotecan. This risk was emphasized by a warning that was added to irinotecan labels in 2005 subsequent to a US Food and Drug Administration (FDA) recommendation, and it has been proposed that cancer patients may be genotyped for *UGT1A1* prior to initiation of irinotecan therapy to enable a preemptive dose reduction for individuals with the UGT1A1*28 allele [13].

In recent years, individualized treatment guided by genotyping has become popular. Mutations involved in the targeting and metabolism of drugs have been highlighted to predict the efficacy and toxicity of treatment. *UGT1A1* polymorphisms contribute to interindividual variability among patients administered irinotecan. Although the proportion of UGT1A1*28 homozygous individuals in the Chinese population is lower than that in Caucasian populations (1−5.5% vs 5−15%) [14–16], the implementation of irinotecan pharmacogenetic testing is increasing in clinical laboratories in China. However, some of these laboratories have only recently adopted clinical pharmacogenetic testing and have limited experience of such techniques. In addition, a variety of methods can be used for irinotecan pharmacogenetic testing, including Sanger sequencing, pyrosequencing [17], high-resolution melting analysis (HRMA) [18], real-time polymerase chain reaction (PCR) [19], and microarray [20]. Each method has its own advantages and limitations. Considering the complexity of these different techniques, it is highly important to standardize testing methods. External quality assessment (EQA) is an essential managerial measure used to assess the proficiency and performance of various *UGT1A1* test methods and laboratories and to identify systematic errors in methodology. To date, there is little experience regarding the quality assurance of *UGT1A1* genotyping in China; to fill in this gap, the Chinese National Center for Clinical Laboratories (NCCL) conducted an EQA in 2015 to evaluate the performance of irinotecan-related genetic testing, including the correct identification of *UGT1A1* genotypes and the subsequent written reports. The College of American Pathologists (CAP) has been conducting irinotecan pharmacogenetic EQA/proficiency testing (PT) since 2007 [21]. In contrast to the CAP PT scheme that uses DNA samples, we used cell samples to simulate clinical samples as these are much easier to prepare and can be used to evaluate the entire testing process. This report is based on EQA data and provides evidence of the excellent analytical performance of *UGT1A1* testing in China.

## Methods

### Preparation of cell samples

UGT1A1*28 allele cell lines and wild-type *UGT1A1* cell lines were prepared. These cell lines (Table 1), purchased from Coriell Cell Repositories (Coriell, New Jersey, USA), consisted of B lymphocytes isolated from human peripheral blood and fibroblast cell line which were immortalized by Epstein–Barr virus. The genetic polymorphisms of these cell lines have been validated by the Genetic Testing Reference Materials (GeT-RM) Coordination Program and can be used for quality assurance, assay development and validation, and proficiency testing [22]. Briefly, lymphoblast cells were cultured in Roswell Park Memorial Institute 1640 medium supplemented with 15% fetal bovine serum, 2 mM L-glutamine, 10 U/mL penicillin, and 10 μg/mL streptomycin (Invitrogen, Carlsbad, USA) at 37°C in an atmosphere of 5% CO_2_; fibroblast cell line (GM17052 in Table 1) were cultured by using a similar method, except that the medium used was Dulbecco’s Modified Eagle Medium. Cells were seeded at approximately 2−5 × 10^5^ viable cells/mL. The time between the creation of subcultures depended on the cell line, but usually occurred at 3−5-day intervals. On the day of cells harvesting, the fibroblast cells were digested with 0.25% trypsin-EDTA and were terminated by medium with 10% fetal bovine serum. Then, both the fibroblast cells and lymphocytes were centrifuged, counted and resuspended in fresh medium to a cell density of 1 × 10^6^ cells/mL. One thousand microliters of each cell line were aliquoted into 1.5-mL vials and labeled.

**Table 1:** EQA panel and the results of genotyping accuracy for the 2015 NCCL/*UGT1A1* EQA survey.

Sample	Coriell Cell Line Number	Coriell Genotype	PCR/Sequencing	No. Correct/Total challenges	Concordance, %	No. error
U1501	GM17248	*28/*28	*28/*28	45/45	100	0
U1502	GM17220	*1/*28	*1/*28	45/45	100	0
U1503	GM16688	*1/*1	*1/*1	45/45	100	0
U1504	GM17289	*1/*1	*1/*1	45/45	100	0
U1505	GM17052	*1/*1	*1/*1	45/45	100	0
U1506	GM17260	*1/*28	*1/*28	44/45	97.8	1
U1507	GM17285	*1/*1	*1/*1	45/45	100	0
U1508	GM17285	*1/*1	*1/*1	45/45	100	0
U1509	GM17285	*1/*1	*1/*1	45/45	100	0
U1510	GM17260	*1/*28	*1/*28	45/45	100	0

### Validation of EQA panel

Samples were validated before distribution by the NCCL reference lab using Sanger sequencing. Genomic DNA was extracted from cell samples using the QIAamp DNA Mini Kit (QIAGEN, Hilden, Germany) according to the manufacturer's instructions. The purity and yield of genomic DNA was assessed using an absorbance-based nucleic acid quantification method (Eppendorf BioPhotometer, Hamburg, Germany). Then, isolated DNA was amplified with specific primers for the UGT1A1*28 gene (forward: 5′AAGTGAACTCCCTGCTACCTT-3′; reverse: 5′-CCACTGGGATCAACAGTATCT-3′) [23]. The UGT1A1 gene was amplified using a standard procedure. Briefly, amplifications were carried out using a Mastercycler (Eppendorf, Humburg, Germany) in a total volume of 50μl, which contained 200ng of genomic DNA, 25μl Gotaq Green Master Mix (Promega, Madison city, USA), 0.2μM of each primer. The following cycling conditions were used: denaturation at 95°C for 5min; followed by 35 cycles of 95°C for 30s, 55°C for 30s and 72°C for 40s; and a final extension of 5min at 72°C.The PCR products were verified by agarose gel electrophoresis and purified, followed by sequencing reactions using a BigDye Terminator v3.1 Cycle Sequencing Kit (Applied Biosystems, Foster City, USA). The sequencing reactions was carried out with an initial denaturing step of 96°C for 1min, followed by 25 cycles of 96°C for 10s, 50°C for 5s and 60°C for 4min. Then, the products were sequenced using an ABI 3500DX Genetic Analyzer (Applied Biosystems). Both the forward and reverse sequencing reactions were performed to exclude PCR-induced errors. All sequencing results were displayed and analyzed with Chromas software and verified through manual inspection. The dispensed cell samples were separately incubated at room temperature (20–25°C) and 2–8°Cfor one week and then analyzed in a stability study.

### Scheme organization

Participation was open to all interested parties in mainland China. Laboratories that are or will be involved in *UGT1A1* genotype testing were particularly welcome. Cell samples were prepared and sent to each participating laboratory. A coded *UGT1A1* EQA panel (n = 10) consisting of four mutant samples and six wild-type samples (Table 1) was used. Each participant was assigned the same samples and was requested to use their own preferred method for DNA extraction and mutational analysis. The test samples were shipped at ambient temperature and delivered to laboratories across the country by Express Mail Service (shipment time is about 1–3 days). Samples were asked to be stored at room temperature or 4°C for no more than two days and temperature under 0°C should be avoided. Participants were encouraged to process the samples as soon as they received to guarantee the quality of DNA extracted The participants were asked to submit their results within 10 days of receiving the test panel. Detailed EQA instructions for the proper handling of specimens were provided in S1 Appendix. Results were reported electronically. For each sample, participants were required to provide a genotype result, the DNA quality (A260/A280 nm ratio) and quantity extracted, and other details of the assay, including information about the number of tests performed each month, genotyping methodology used, and laboratory accreditation/certification status.

### Scoring of the reports

Each participating laboratory were asked to provide a detailed written report for the first sample “U1501” as they would normally do on a routine basis. The assessment of the written reports was not taken into account in deciding successful participation in the EQA scheme, but was used for educational purposes only. We defined 15 essential items that should be present in a good report for *UGT1A1* genotyping (Table 2) based on the International Organization for Standardization (ISO) 15189:2012 requirements for medical laboratories [24]. The content in the written report of individual laboratory was analyzed and scored. One point was awarded when an item was present and correct. No points were awarded if an item was incorrect or absent.

**Table 2:** Different items used for scoring of reports of the 2015 NCCL/*UGT1A1* EQA survey.

Item Description
1 Sampling/arrival date • The date and time of sample collected
2 Sample identifier
3 Date of report
4 Signature
5 Unique identifier on each page • For example, by lab identifier, name. . .
6 Total pages • Page 1 of 2, 1/2 (not 1,2,3,. . .)
7 Consultants • Lab address and phone number
8 Nature of the sample • The nature of sample collected or sample source (e.g., peripheral blood, cells, biopsies. . .)
9 Reason for testing
10 Genotype
11 Interpretation of the results • Comments/results and conclusion,. . .
12 List of alleles tested • The alleles which the labs can detect
13 Method used
14 Report title • Refers to UGT1A1 genotyping and clearly distinguished from other reports
Refers to therapy • Dosing recommendations

### Data analysis

The reported genotyping results were compared with the genotype verified by the reference laboratory. Two genotyping error types were used to evaluate the results: false-negative results (identification of the wild-type instead of a mutation or identification of an incorrect mutation) and false-positive results (identification of a mutation instead of the wild-type). If a data set had a minimum of 80% correct responses, it was considered to be proficient. Genotyping accuracy, types of errors, analytical sensitivity, and specificity were computed.

All analyses were performed using the MEDCALC software (MedCalc Software, Mariakerke, Belgium). Comparison of rates was performed by Fisher’s exact test. A P value of <0.05 was considered to indicate statistical significance. Confidence intervals of 95% (CI 95%) were determined.

## Results

### Sample validation

Before distribution, each cell sample was verified by Sanger sequencing designed to detect specific alleles (Table 1). The results from the reference laboratory evaluation indicated that the panel performed as expected. The genotype concordance of *UGT1A1* between Coriell validation and Sanger sequencing was 100%. The genomic DNA yields were >10 μg per sample and were adequate for *UGT1A1* genetic testing. Moreover, the data obtained from stability analyses revealed that after incubation for seven days at room temperature, more than 10μg DNA could be extracted from each cell sample; and after incubation for seven days at 2–8°C, the minimum amount of DNA extracted were 5μg from each sample. The stability study demonstrated that the amount of DNA was sufficient for downstream analysis.

### Participating groups and methodologies

Forty-five laboratories, including 31 hospital laboratories and 14 commercial laboratories/reagent manufacturers, participated in this national *UGT1A1* EQA scheme in 2015 and submitted their results within the requested time frame. The average number of samples tested per month by the participants was 13 (range: 1–100). Twenty-eight laboratories (62.2%) analyzed less than 10 samples per month. Fourteen of the 45 participating laboratories (31.1%) were accredited. More specifically, 12 laboratories (26.7%) were accredited according to ISO 15189 or ISO 17025, and the other two laboratories (4%) were certified according to CAP.

Participants used a variety of techniques to perform *UGT1A1* genotyping. The most frequently used methodology was pyrosequencing (26/45, 57.8%), followed by PCR-capillary electrophoresis (CE) (6/45, 13.3%), Sanger sequencing (5/45, 11.1%), next-generation sequencing (NGS) (3/45, 6.7%), PCR-microarray (2/45, 4.4%), real-time PCR (1/45, 2.2%), HRMA (1/45, 2.2%), and matrix-assisted laser desorption/ionization mass spectrometry (MALDI-TOF-MS) (1/45, 2.2%). Thirty-one of the 45 laboratories (68.9%) used commercial kits developed by manufacturers for research purposes or for use in clinical diagnostics, and 14 participants (31.1%) used laboratory-developed methods. Among those using commercial kits, the commercially available assay manufactured by QIAGEN was the most widely used (n = 22, 48.9%); four other commercial kits were used among the remaining nine participants.

### UGT1A1 genotyping performance

We received 45 completed data sets. No laboratories reported any problems with DNA extraction from the cell samples. The median quality of extracted DNA was desired with an average A260/280 of 1.83 (range from 1.71 to 2.06), and the mean DAN quantity was 13.7μg (range from 4.8 to 36.1μg), which were similar to the expected value and was sufficient for downstream analysis. The test results of each sample were compared with the expected genotypes verified by the reference lab (Table 1). In total, 44 participants reported all genotype results correctly, and only one genotype mistake was reported in one data set. No difference in genotyping accuracy was observed between the designation of the wild-type *UGT1A1* alleles and any of the variant alleles (P = 0.221). We compared the performance of *UGT1A1* genotyping among the different participating groups. The results revealed that there was no statistically significant difference in genotyping accuracy between hospital laboratories and commercial laboratories/reagent manufacturers (P = 0.501) or between accredited and non-accredited laboratories (P = 0.523). There were no false-positive results and only one false-negative result, which incorrectly identified UGT1A1*1/*28 as the wild-type for sample U1506. It should be noted that the false-negative result was produced by a non-accredited laboratory.

The proficiency of *UGT1A1* genotyping is summarized in Table 3. Surprisingly, all 45 participants met the criteria for passing this EQA. Forty-four data sets (99.7%) were found to be 100% proficient (i.e. with all genotypes detected correctly). Furthermore, the analytical sensitivity and specificity of the individual testing methodologies were determined (Table 3); both the laboratory-developed assays and commercial kits showed excellent analytical performance, with analytical mistakes occurring very rarely. Both the overall sensitivity and specificity of all techniques were high [99.4% (179/180 challenges) and 100% (270/270 challenges), respectively] in this EQA survey.

**Table 3:** Proficiency results and characteristics of genotyping methods used in the 2015 NCCL/*UGT1A1* EQA survey.

Assay	No. of data sets	No. of data sets proficient at*:				*UGT1A1* genotypes	
		100%	99–90%	89–80%	<80%	Sensitivity(%;CI 95%)	Specificity(%;CI 95%)
						Correct mutation/total mutation challenges	Correct wild-types/total
							wild-type challenges
Pyrosequencing-QIAGEN	22	21	1	0	0	98.86;93.83–99.97 (87/88)	100;97.24–100 (132/132)
Pyrosequencing-Sanji	2	2	0	0	0	100;63.06–100 (8/8)	100;73.54–100 (12/12)
In-house Pyrosequencing	2	2	0	0	0	100;63.06–100 (8/8)	100;73.54–100 (12/12)
Real-time PCR skybiotech	1	1	0	0	0	100;39.76–100 (4/4)	100;54.07–100 (6/6)
PCR-CE YUANQI BIO	5	5	0	0	0	100;83.16–100 (20/20)	100;88.43–100 (30/30)
In house PCR-CE	1	1	0	0	0	100;39.76–100 (4/4)	100;54.07–100 (6/6)
In-house NGS	3	3	0	0	0	100;73.54–100 (12/12)	100;81.47–100 (18/18)
In-house sanger sequencing	5	5	0	0	0	100;83.16–100 (20/20)	100;88.43–100 (30/30)
In-house PCR-microarray	2	2	0	0	0	100;63.06–100 (8/8)	100;73.54–100 (12/12)
In-house MALDI-TOF-MS	1	1	0	0	0	100;39.76–100 (4/4)	100;54.07–100 (6/6)
HRMA-Szwz	1	1	0	0	0	100; 39.76–100 (4/4)	100; 54.07-100(6/6)
All assay	45	44	1	0	0	99.44;96.94–99.99	100;98.64–100
						(179/180)	(270/270)

*100% proficient: all genotype detected correctly. 80%– 99% proficient: 80%– 99% of genotype detected correctly. < 80%: < 80% of genotype detected correctly.

PCR, polymerase chain reaction; CE, capillary electrophoresis; NGS, next generation sequencing; MALDI-TOF-MS, matrix-assisted laser desorption/ionization time of flight mass spectrometry; HRMA, high-resolution melting assay.

### Reporting of results

**Fig 1:**
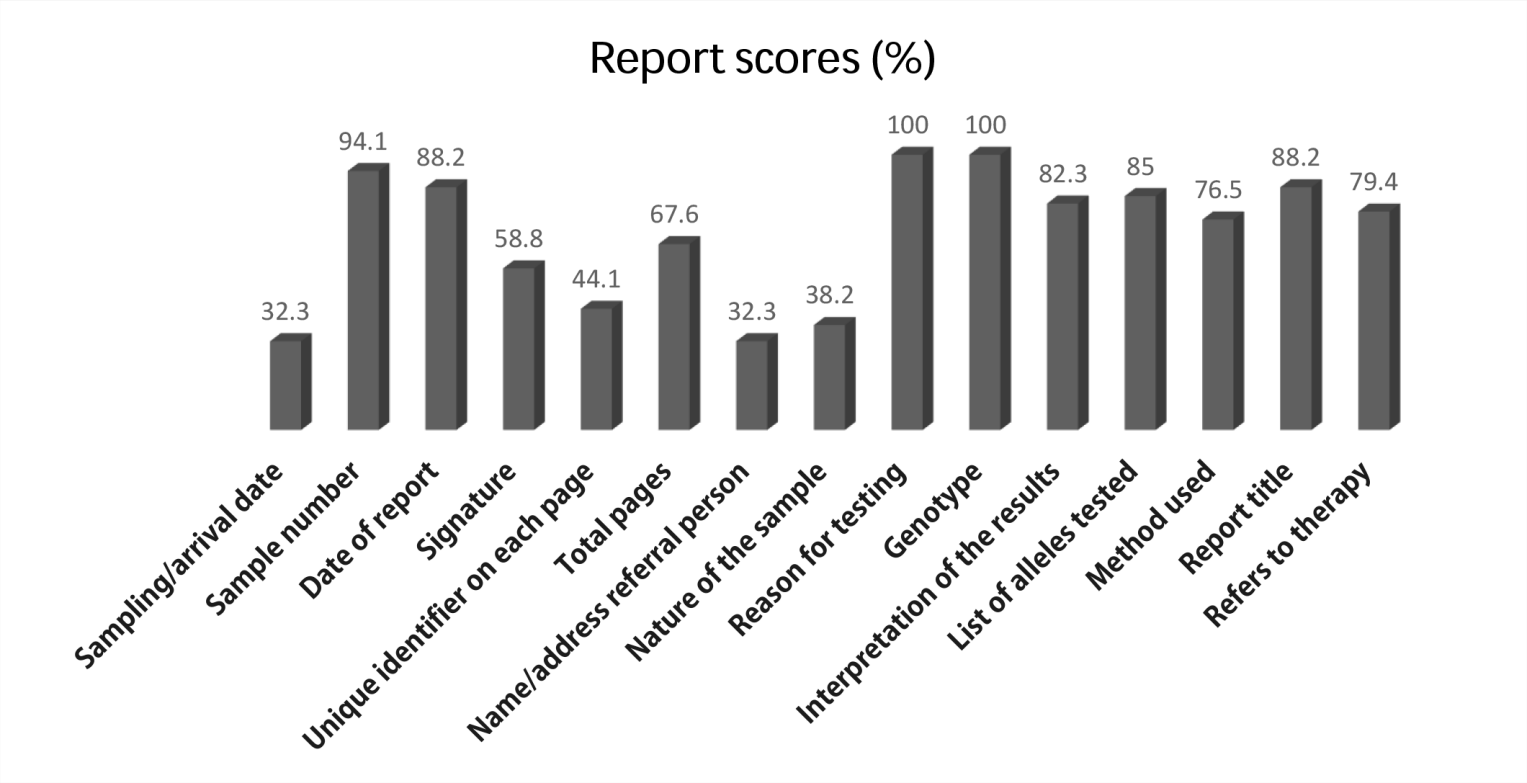
Scores of different report items of the 2015 NCCL/*UGT1A1* external quality assessment survey, n = 34 reports analyzed.

Reports for sample U1501 were sent by 34 of the 45 participating laboratories. The mean score of the reports was 10.8 points (out of a maximum of 15), ranging from 3 to 15 points. Fig 1 describes the presence of the 15 items in the laboratories’ written reports. According to the written report, the time-lag between sample preparation in EQA laboratory and arrival at participating laboratories was 1–3 days. A review of these reports showed that a number of laboratories did not include elements that were considered crucial for an informative report. Critical elements such as interpretation of the results, list of alleles tested, and test methodology used were missing in 18.2%, 15%, and 23.5% of the reports, respectively. Items usually included in the reports were the genotype, reason for testing, sample number, data of the report, and total pages. Sampling/arrival date and name/address of the referring clinician were missed in the reports of the majority of laboratories. Other items that were often not included in the reports were signatures, unique identifier on each page, and the nature of the sample.

### Overall assessment and participant feedback

The DNA extraction process and the written report were evaluated for education purpose in the first pilot EQA, and the genotyping results were used to evaluate the laboratory performance. Because the known genotyping results for each of the sample were validated in the NCCL reference laboratory, we compared the consistency of a given participant’s results against the reference results. Based on the consistency, the mean genotyping score were classified as competent (100% correct responses), acceptable (>80% correct responses), or improvable (<80% correct responses). Of the 45 data sets, the performance were found to be competent in 44 analysis, and one data set met the criteria with acceptable results. In other words, all the laboratories achieved proficient performance in this EQA scheme.

On completion of the evaluating process, the EQA provider then compiled the data, and a full detailed scheme feedback report was made available to all scheme participants. Summarized results and educational insights for the pilot scheme were included in the feedback (S2 Appendix).

## Discussion

China has a high incidence of colorectal cancer with approximately 253,000 new cases diagnosed each year, with an upward trend in recent years [25]. Irinotecan is used in first-line chemotherapy with 5-fluorouracil and oxaliplatin for the treatment of metastatic colorectal cancer. However, delayed diarrhea and neutropenia caused by irinotecan may occur in some patients during or after treatment, subsequently affecting the patient’s quality of life. Pharmacogenetic testing for *UGT1A1* polymorphisms is widespread in China and can help improve patient care by indication of an appropriate individualized irinotecan therapy. The success of implementing clinical irinotecan pharmacogenetic testing into clinical practice largely depends on the accuracy of genotyping; thus, quality assurance is critical, especially for laboratories in China that have only recently started to perform molecular diagnostics. Here, we described a study performed in mainland China that aimed to investigate the laboratory performance of *UGT1A1* genetic testing by using an EQA survey.

According to the EQA program, the results from this study showed that the overall genotype concordance of *UGT1A1* genetic testing was excellent and involved few genotyping errors. All the participating laboratories met the criteria for the accurate detection of the *UGT1A1* genotype. Strictly speaking, any errors are clearly unacceptable in clinical practice and quality control. It is satisfactory that no false-positive results were found in this EQA program and only one laboratory incorrectly reported UGT1A1*1/*28 as the wild-type genotype (false negative). In the laboratory diagnosis process, false-negative results may occur because of limitations of the methodology or poor laboratory performance; a validation test should be conducted before implementing one methodology into routine practice. In our study, the false-negative result may have been related to laboratory performance. On one hand, the other 22 labs using the same genotyping kit (QIAGEN) reported the correct genotype, on the other hand, samples U1502 and U1510, which possessed the same polymorphism, were all correctly detected. This error was likely clerical in nature and emphasized the need for internal quality control. Further, this false-negative result would cause the treatment of the cancer patient with a high risk of side effects. It has been reported that UGT1A1*28 heterozygotes and homozygotes have an increased risk of irinotecan-related severe diarrhea (33.0% and 70.0%, respectively) compared to wild-type individuals (17.0%) [12]. In this respect, both false-negative and false-positive results are potentially harmful for patients; thus, regular quality control is essential.

Another important issue in *UGT1A1* genotyping is the method used for testing. We used Sanger sequencing as a validation method, as it can identify all possible mutations in the analyzed gene segment and is widely acknowledged as the standard for the direct detection of sequence variants. We noticed that PCR-sequencing (including pyrosequencing, Sanger sequencing, and NGS) was the most widely used strategy for the detection of polymorphisms, and was used by 34 of the participating laboratories (75.6%). All of these laboratories achieved an excellent performance. It is worth noting that three participants used a laboratory-developed test based on NGS. NGS is a rapid, highly developed technology with a wide range of potential applications in clinical laboratories and with benefits including increased technological capacity and decreased costs. However, most laboratories generally do not have much experience with this method, and it is necessary to introduce laboratory standards for NGS clinical tests. In addition, almost all testing methods adopted in this EQA performed with good analytical sensitivity and specificity. Although the laboratory-developed test acquired a hit rate of 100% accuracy, we cannot conclude that the assay is superior because of the low number of participants who used this assay. Compared to laboratory-developed tests, commercial kits contain both positive and negative controls and are easier to standardize. Therefore, it is advisable for laboratories with little experience in molecular detection to use such kits.

Proper reporting of results is another important step in clinical pharmacogenetic testing. Our EQA program included an evaluation of the reporting of results. A clear and complete written report is very important to correctly provide diagnostic results so that the referring clinicians have accurate and comprehensive information available to help them make the best clinical therapeutic decision. The overall quality of the reports in this EQA survey was not good; 11 of the 45 clinical laboratories (24.4%) did not submit reports, and reports that were submitted often lacked essential information. Missing elements in the report could provide insufficient or even misleading information. For instance, nine laboratories (25%) did not list the alleles tested. If negative results were reported, it is very important to know which alleles were tested for and which were not; thus, those alleles that were tested for should be listed. Furthermore, the interpretation of results was very diverse. Some reports (6/33, 18.2%) did not provide an interpretation of the results and stated only the genotype result. Most reports (25/33, 75.8%) recommended a reduction of the dosage when using irinotecan or a change to another medicine. One laboratory interpreted the genotyping result according to the Dutch Pharmacogenetics Working Group guideline for irinotecan and *UGT1A1* [26]. In addition, there were two laboratories which advised against using irinotecan. However, the US FDA has advised a lower starting dose by at least one level of irinotecan in UGT1A1*28 homozygous patients to reduce the risk of adverse drug events. The variation in result interpretations may be related to the lack of any criteria for genotype-guided irinotecan therapy in China. The variation in the format of the reports highlighted the need for standardization and clarity of report content. More education on reporting test results is needed in order to promote good laboratory reporting practices. The laboratories were recommended to include the 15 essential items that listed in Table 2 in their future diagnostic report. In addition, based on the US FDA’s approval and the guideline for the irinotecan and *UGT1A1* which published in Dutch and French [13, 26, 27], it is recommended that the UGT1A1*28 diagnostic report should emphasize on reducing initiation dose of irinotecan for patients with the UGT1A1*28/*28 genotype to lower the risk of irinotecan related toxicity, and no dose adjustment is needed for patients with UGT1A1*1/*28 and UGT1A1*1/*1. Feedback involving the detailed data analysis conducted on the EQA results was subsequently sent to the participating laboratories so that they were aware of the performance of various test methods and laboratories in the assessment.

The genotype accuracy of *UGT1A1* in this EQA (449 responses/450 challenges, 99.44%) was superior to that in the CAP pharmacogenetic testing survey (586 responses/614 challenges, 95.43%) [21]. This result may be because regulations regarding the laboratory environment and operation procedures for laboratories performing PCR-based tests were introduced in China in 2002. In order to provide a true assessment of the quality of laboratory performance, it is important that the complete testing process be assessed. The CAP PT used DNA samples in laboratories and did not evaluate the entire process, as it was difficult to obtain an adequate volume of previously characterized whole-blood samples. However, we used cell samples instead of DNA to assess the whole genotyping process and to ensure a closer relation between the EQA and routine clinical activity. In this respect, our approach was creative since we used material closely resembling that used in clinical practice. The DNA extraction process can be affected during the collection, transport, and storage of samples, and downstream analysis in molecular diagnostic assays would be affected by poor DNA quality. Since DNA extraction is an important issue in DNA-based analysis, special attention should be paid. Generally, the best way to check the entire DNA extraction process is based on blood specimen because it represents what is actually tested in clinical practice. The EQUAL project in European and the SPIDIA project in Italy have conducted series of comprehensive and rigorous EQA scheme to standardize the processing of DNA/RNA extraction in blood samples, which provided a reference of guideline for the handling blood samples [28–30]. By contrast, the assessment of DNA extraction in our EQA pilot scheme is preliminary. The NCCL will develop EQA schemes with more stringent and comprehensive conditions to evaluate the DNA extraction process. However, it is usually hard to obtain sufficient appropriate whole-blood specimen for the evaluation of performance of a specific diagnostic test. Under this circumstance, cell samples could be a surrogate for blood samples because of easy to prepare and expand.

Our study only evaluated the genotyping of UGT1A1*28 polymorphism, however, there are other *UGT1A1* variants such as UGT1A1*6 (211G>A, G71R), UGT1A1*36 (five TA repeats), and UGT1A1*37 (eight TA repeats), which are known to be important for UGT1A1 enzyme function. Nevertheless, UGT1A1*36 and UGT1A1*37 occur almost exclusively in populations of African origin and are rarely in Asians [31], the pharmacogenetic laboratories in China have not implemented the detection of UGT1A1*36 or UGT1A1*37 into clinical practice. UGT1A1*6 reduce catalytic function by 60% in homozygotes and is most frequent among Asians (13.0–23.0%) [32, 33]. The combination test of UGT1A1*6 and UGT1A1*28 may prove to be a potential predictive biomarker of irinotecan-induced severe neutropenia in the Asian population [9]. However, a survey concerning of irinotecan-related *UGT1A1* genotyping was conducted before this EQA scheme and showed that less than 20% participating laboratories developed UGT1A1*6 detection to date. With UGT1A1*6 being found more commonly for clinical use, the genotyping of UGT1A1*6 would also be established in many laboratories in China. In the future, we plan to include UGT1A1*6 genotyping in the PT scheme for irinotecan pharmacogenetic testing.

The main objective of an EQA scheme is to improve molecular-diagnostic-testing quality, establish interlaboratory consistency, and identify potential laboratory issues. Furthermore, it is a useful tool to help clinicians to pay more attention to both the test results and the entire testing process. The limitations of genetic-testing quality summarized here are common issues in routine laboratory diagnosis and provide significant experience-based advice to other laboratories wanting to carry out similar pharmacogenetic tests in the future.

## Conclusion

In conclusion, the clinical laboratories in this study demonstrated excellent analytical sensitivity and specificity in *UGT1A1* genotyping. Continuous EQA and education regarding written reports is needed to improve and guide the safety and efficacy of irinotecan administration for cancer patients. In the future, we aim to expand this program to include more laboratories to provide a baseline picture of the quality analysis of *UGT1A1* testing in China.

## Supporting Information

S1 Appendix EQA scheme instructions for participants. (PDF)

S2 Appendix The feedback to the participants. (PDF)
