# Supplementary material for: Molecular Genetics External Quality Assessment Pilot Scheme for Irinotecan-Related UGT1A1 Genotyping in China
Source: PLoS One. 2016 Jan 28;11(1):e0148081. doi: 10.1371/journal.pone.0148081 (PMC4731084; doi:10.1371/journal.pone.0148081)
Supplement: S1 Appendix — (PDF) [file pone.0148081.s001.pdf]

Instructions

Ironotecan related *UGT1A1* genotyping EQA pilot scheme

1. EQA panel information

The EQA panel contained 10 cell samples: **U1501-U1510**. The cell lines harboring common *UGT1A1* polymorphisms were immortalized from human peripheral blood. Each vial contains 1.0 mL of suspended cells at a density of 1×10^6 cells/mL.

NOTE:

-Even though the samples were tested negative for pathogenic, all samples should be treated as infectious specimen for unknown biological risk.

-Please check if the number of samples and the quantity of specimen matched with this EQA after sample receipt.

2. What do you do with the samples?

DNA extraction:

Please process the cell samples for DNA extraction as soon as sample receipt. Samples can be stored at room temperature or 4 for no more than two days. Please do not store the samples under 0℃. DNA quality **(A260/A280)** and quantity should be measured.

UGT1A1 polymorphisms detection:

Please use the standard procedure under routine handling conditions in your laboratory.

3. What and how do you send your results back to NCCL?

Data results

Report the results in the scheduled result form. Please log in the public email to get the "Result Form" to report the results. Email address: cyp2c19cn@163.com; Password: 201501.

Written report

Provide a detailed written report for sample "U1501" as you would normally do on a routine basis. (Format of PDF, Word, JPEG… are allowed).

Ways to return results:

Your filled result form and written report should be submitted to NCCL via e-mail.

E-mail address: linpgx@163.com

NOTE:

-The **deadline** for results submitting is **February 10th, 2015**. Results received after the deadline would not be included in statistical analysis.

If there were any professional questions about the EQA activity, please contact with the institution of NCCL.

Contacts: Jinming Li, Guigao Lin.
Address: Beijing Hospital, No.1 Dahua Road, Dongdan, Beijing, People’s Republic of China.
Tel: +86 10 58115053
