# Supplementary material for: Molecular Genetics External Quality Assessment Pilot Scheme for Irinotecan-Related UGT1A1 Genotyping in China
Source: PLoS One. 2016 Jan 28;11(1):e0148081. doi: 10.1371/journal.pone.0148081 (PMC4731084; doi:10.1371/journal.pone.0148081)
Supplement: S2 Appendix — (PDF) [file pone.0148081.s002.pdf]

Summary of the Molecular Genetics External Quality Assessment Pilot Scheme for Irinotecan-related UGT1A1 genotyping in 2015

Organizer: National Center for Clinical Laboratories.

Contacts: Jinming Li, Guigao Lin.

Address: Beijing Hospital, No.1 Dahua Road, Dongdan, Beijing, People's Republic of China.

Tel: +86 10 58115053

Fax: +86 10 65212064

1. Aim of this EQA scheme

The implementation of irinotecan related pharmacogenetic testing is increasing in clinical laboratories in China. The purpose of this scheme was to monitor the quality of *UGT1A1* testing and improve the quality by providing a means for evaluation and education of laboratories.

2. General character of this EQA scheme

A total of 45 laboratories participated in this EQA scheme. The EQA panel included 10 cell samples concerning of *UGT1A1**1/*1, *UGT1A1**1/*28, and *UGT1A1**28/*28. Each sample contained one thousand microliters of each cell lines diluted with fresh medium to a density of 1×10^6 cells/mL. Laboratories were asked to proceed the EQA samples as they usually do in routine basis.

3. The expected results and scoring principles

Table 1. EQA panel for the 2015 NCCL/*UGT1A1* EQA survey

**Sample**	U1501	U1501	U1501	U1501	U1501	U1501	U1501	U1501	U1501	U1501
**Genotype**	*28/*28	*1/*28	*1/*1	*1/*1	*1/*1	*1/*28	*1/*1	*1/*1	*1/*1	*1/*28

The genotyping concordance was determined against the genotype verified by the reference laboratory. The criteria used for considering a data set proficient is at least 80% genotype accuracy: (correct challenges/total challenges) ×100%.

4. EQA performance of participants

Table 2. The results of genotyping accuracy for the 2015 NCCL/*UGT1A1* EQA survey

Sample	Genotype	No. Correct/Total challenges	Concordance, %	No. error

U1501	*28/*28	45/45	100	0
U1502	*1/*28	45/45	100	0
U1503	*1/*1	45/45	100	0
U1504	*1/*1	45/45	100	0
U1505	*1/*1	45/45	100	0
U1506	*1/*28	44/45	97.8	1
U1507	*1/*1	45/45	100	0
U1508	*1/*1	45/45	100	0
U1509	*1/*1	45/45	100	0
U1510	*1/*28	45/45	100	0

Table 3. Errors of genotyping in the 2015 NCCL/*UGT1A1* EQA survey

Sample	Methodology	Expected genotype	Reported result
U1502	Pyrosequencing	*1/*28	*1/*1

5. Analysis of methodology

Table 4. Proficiency results and characteristics of genotyping methods used in the 2015 NCCL/*UGT1A1* EQA survey

Assay	No. of data sets	No. of data sets proficient at*:			
		100%	99–90%	89–80%	<80%
Pyrosequencing-QIAGEN	22	21	1	0	0
Pyrosequencing-Sanji	2	2	0	0	0
In-house Pyrosequencing	2	2	0	0	0
Real-time PCR skybiotech	1	1	0	0	0
PCR-CE YUANQI BIO	5	5	0	0	0
In house PCR-CE	1	1	0	0	0

In-house NGS	3	3	0	0	0
In-house sanger sequencing	5	5	0	0	0
In-house PCR-microarray	2	2	0	0	0
In-house MALDI-TOF-MS	1	1	0	0	0
HRMA-Szwz	1	1	0	0	0
All assay	45	44	1	0	0

*100% proficient: all genotype detected correctly. 80% – 99% proficient: 80% – 99% of genotype detected correctly. < 80%: < 80% of genotype detected correctly.

PCR, polymerase chain reaction; CE, capillary electrophoresis; NGS, next generation sequencing; HRMA, high-resolution melting assay; MALDI-TOF-MS, matrix-assisted laser desorption/ionization time of flight mass spectrometry.

6. Summary and recommendations

All the 45 participating laboratories met the criteria for the accurate detection of the *UGT1A1* genotype. Due to confidentiality principle, this report does not reflect performance of individual participants. Most clinical labs used commercial available kits, and part of labs used laboratory-developed test (LDT). The cell samples were processed without methodology limitation in this EQA, however, the labs were asked to use the routine assay which aims that the result of the quality evaluation reflecting the actual detection ability of the labs. In general, the sensitivity and specificity of each assay were satisfied. However, the LDT assays needed to be verified and/or validated by using reference materials or quality control product before implementing for clinical purpose.

In the EQA scheme, labs were asked to submit a written report for sample “U1501”. However, about a third of the labs didn’t submit the report, and some labs submit the original data instead. Based on the ISO15189 standard and a guideline document regarding reporting of molecular results in general, many reports showed a shortage of information. Critical elements such as laboratory identifier, the nature of sample, date

of specimen collection, test methodology used, list of alleles tested, and the interpretation of the results. The result is the crux of every report. It is recommended that a clinical interpretation involves describing what the result means for the patient, either in general or based on specific knowledge of that patient's situation, should present. In the pharmacogenetic testing, the written report concerning of the clinical significance and medicine recommendation is preferred. Based on the FDA's approval and the guideline for the irinotecan and *UGT1A1* published in French and Dutch, we recommend the *UGT1A1**28 diagnostic report for each possible genotype should emphasize to reduce initiation dose of irinotecan for patients with the *UGT1A1**28/*28 genotype, and no dose adjustment is needed for patients with *UGT1A1**1/*28 and *UGT1A1**1/*1. Alternatively, one should reference a reliable Website or publication at which generic test information is found.

In total, the participating labs have the ability to provide a reliable testing result in *UGT1A1* testing. The education in written reports is needed to improve. The labs can adopt remedial measures in their clinical practice refer to this summary.

Reference:

1. ISO15189: 2012, Medical laboratories-requirements for quality and competence. ISO/TC, 2012. Available: http://www.iso.org/iso/catalogue_detail?csnumber=56115
2. Gulley ML, Braziel RM, Halling KC et al. Clinical laboratory reports in molecular pathology. Arch Pathol Lab Med 2007;131:852–863.
